# Teaching through their eyes: effects on optometry teachers’ adaptivity and students’ learning when teachers see students’ gaze

**DOI:** 10.1007/s10459-024-10325-3

**Published:** 2024-04-10

**Authors:** Robert-Jan Korteland, Ellen Kok, Casper Hulshof, Tamara van Gog

**Affiliations:** Department of Education, Utrecht University, P.O. Box 80140, Utrecht, 3508 CS Netherlands

**Keywords:** Adaptive teaching, Eye tracking, Optical coherence tomography, Optometry, Student-to-teacher gaze displays, Teacher support adaptivity

## Abstract

Adaptive teacher support fosters effective learning in one-to-one teaching sessions, which are a common way of learning complex visual tasks in the health sciences. Adaptive support is tailored to student needs, and this is difficult in complex visual tasks as visual problem-solving processes are covert and thus cannot be directly observed by the teacher. Eye-tracking apparatus can measure covert processes and make them visible in gaze displays: visualizations of where a student looks while executing a task. We investigate whether live dynamic gaze displays help teachers in being more adaptive to students’ needs when teaching optical coherence tomography interpretation in one-to-one teaching sessions and whether this fosters learning. Forty-nine students and 10 teachers participated in a one-to-one teaching session in clinical optometry. In the control condition, teachers saw the learning task of the student and could discuss it with them, whereas in the gaze-display condition, teachers could additionally see where the student looked. After the 15-minute teaching session, a test was administered to examine achievement. Furthermore, students filled in the ‘questionnaire on teacher support adaptivity’, and teachers rated how adaptive their support was. Bayesian analyses provide some initial evidence that students did not experience support to be more adaptive in the gaze-display condition versus the control condition, nor were their post-test scores higher. Teachers rated their provided support as being more adaptive in the gaze-display versus the control condition. Further research could investigate if live dynamic gaze displays impact adaptive teaching when used over longer periods or with more teacher training.

**Supplementary Information:**

The online version contains supplementary material available at 10.1007/s10459-024-10325-3.

## Introduction

**Fig. 1:**
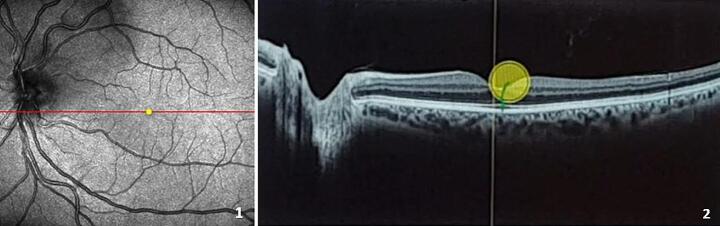
Example of an OCT Image of a Normal Retina. *Note* (1) Fundus photograph (2) OCT image: The circle represents the gaze location of a student looking at the OCT image

One-to-one teaching sessions are central to learning complex visual tasks in medicine (Lyons et al., 2017), such as interpreting fundus photographs or optical coherence tomography images (OCTs) in clinical optometry. OCT is a standard technology used in primary care for routine cross-sectional digital imaging of the human retina (Fig. 1). Optometry students must learn to work with the equipment and diagnose diseases using this imaging technique, which generally takes place in one-to-one teaching sessions with an expert (teacher). While highly resource-intensive, such teaching is still widely applied in health sciences education, in particular in internships and residency training (Butler et al., 2019; Hari et al., 2022). High-quality teaching, using adaptive support, might make the most optimal use of those one-to-one teaching moments.

However, there is variation in the teaching quality, which might impact student learning (Ben-Sasson et al., 2019; Konishi et al., 2020; Minshew et al., 2022; Woolley & Jarvis, 2007). Good teachers provide adaptive support (Van de Pol et al., 2015), that is tailored to the learners’ academic ability (Van de Pol & Elbers, 2013). Adaptive support requires teachers to adequately estimate what the student does or does not understand and provide the right amount of challenge or support (Van de Pol & Elbers, 2013). Estimating student knowledge in teaching complex visual tasks might be particularly difficult for teachers as visual problem-solving processes are covert and thus cannot be directly observed (Kok, 2016). For example, when a student spots pathological changes in an OCT image and the teacher asks in which retinal layer the change occurs, students often confuse terminology for outer and inner layers (Dolz-Marco, 2020). Moreover, a student could be guessing, giving the correct answer while looking at the wrong layer, leading the teacher to overestimate the student’s level of knowledge and provide less support (Van de Pol et al., 2022), hampering the student’s learning.

With eye-tracking technology, covert viewing behavior can be made visible by gaze displays (see Fig. 1). Making this covert viewing behavior visible to teachers might allow them to provide more adaptive support. There is increasing evidence that teachers can use gaze displays to understand learning processes (Emhardt et al., 2022; Kok et al., 2023) and that they are willing and able to use gaze displays to improve their teaching (Knoop-van Campen et al., 2021). In this study, we investigated if showing gaze displays to teachers during a one-to-one teaching session can help them provide more adaptive support and if this, in turn, leads to better student performance.

### Teacher adaptive support for learning complex visual tasks

Teachers’ adaptive support fosters effective learning (Van de Pol et al., 2022). It requires estimating the student’s academic needs and then providing the appropriate amount of support given those needs (Van de Pol & Elbers, 2013). Van de Pol et al. distinguish between four facets of (non)adaptive support (2022). Adaptive support means increasing teacher regulation upon low student understanding, for example, by providing more guidance and examples (Adaptive + or A+). It is also adaptive if teachers decrease regulation when students show high understanding (Adaptive- or A-). Non-adaptive teachers, in contrast, increase regulation upon higher understanding (Non-adaptive + or NA+), for instance, pointing at the correct retinal layer while the student already knows what to focus on. Finally, non-adaptive teachers can decrease regulation upon lower understanding (Non-adaptive- or NA-), for example, by telling the student to execute the complete learning task individually while the student is struggling. Thus, to provide adaptive support, teachers have to accurately judge students’ understanding and adapt to it by increasing or decreasing regulation.

Adaptive support is difficult to provide because teachers often overestimate students’ understanding. Consequently, the support they provide or withhold is not always tailored to student needs and therefore not always effective for learning (Südkamp et al., 2012). In learning complex visual tasks, it is even more difficult for teachers to provide adaptive support, as the students’ problem-solving processes are mostly covert and thus cannot be directly observed by the teacher (Cox, 2002). Moreover, people know surprisingly little about their visual behavior and cannot report where they have looked (Kok et al., 2017) or what search strategy they executed (Aizenman et al., 2017). Since it is difficult for students to report on their covert viewing behavior and for teachers to observe it directly, technology to visualize viewing behavior (i.e., eye-tracking technology) might help teachers in providing adaptive support.

### Eye-tracking methodology and gaze displays

Eye-tracking methodology can be used to record covert viewing behavior (Jarodzka et al., 2017; Kok & Jarodzka, 2017) and visualize it to teachers so they can adapt their teaching based on the information they can infer from those visualizations. In 1967, Yarbus already argued that “eye movements reflect the human thought process; so the observer’s thought may be followed to some extent from the records of eye movements.” (Yarbus, 1967, p. 190) Visualizations of recorded eye movements are called gaze displays (Van Wermeskerken et al., 2018). Gaze displays have several applications in (medical) education (Scheiter & Van Gog, 2009; Van Gog & Jarodzka, 2013; Van Gog & Scheiter, 2010; Van Gog et al., 2009) for example, in the form of eye-movement modeling examples (Emhardt et al., 2022; Seppänen & Gegenfurtner, 2012) or as a feedback instrument (Kok et al., 2022).

A novel application of gaze displays, is to help teachers in providing more adaptive support. For example, when teaching OCT interpretation, if the student looks at the outermost layer of the retina while speaking about the innermost layer and the teacher sees where the student is looking, they can spot this misconception and adaptively support the student in finding the correct layer.

An important prerequisite for teachers’ use of gaze displays to adapt teaching is that teachers can indeed infer the underlying cognitive processes from the gaze displays (Kok et al., 2023). Several studies have shown that people can interpret gaze displays in terms of perceptual and/or cognitive processes (Bahle et al., 2017; Emhardt et al., 2020; Foulsham & Lock, 2015; Greene et al., 2012; Van Wermeskerken et al., 2018; Zelinsky et al., 2013). For example, Emhardt et al. (2020) presented participants with gaze displays of observers who interpreted line graphs and answered a multiple-choice question about the graph. Based on displays of the observers’ gaze, participants could guess, at above-chance levels, which of the four answer options was selected by the observer. Likewise, secondary education teachers could infer strategic reading behaviors from gaze displays of students reading multimedia texts (Knoop-van Campen et al., 2021). Teachers also reported appreciating the insights gained from seeing learners’ gaze displays (Špakov et al., 1995; Knoop-van Campen et al., 2021).

Those studies show the potential of gaze displays to help adaptive teaching in one-to-one teaching sessions in medical education. However, it is yet unknown whether those findings generalize to this context and, more specifically, whether *live* gaze displays can help teachers to provide adaptive support (and thus foster learning) in one-to-one teaching sessions. In earlier studies, gaze display interpretation was not performed under time pressure, whereas in one-to-one sessions, teachers must interpret gaze displays quickly to interact with the information and adapt their teaching if necessary. Additionally, earlier studies did not yet investigate to what extent teachers’ provided adaptive support, and what the effects on learning are, which is necessary to find out whether they achieve the intended goal.

Thus, our aim was to investigate whether teachers provide more adaptive instructional support in one-to-one teaching sessions when they see the student’s gaze displayed in real-time and whether that benefits students’ learning and their perception of teacher support. To investigate whether teacher support is more adaptive when they see student’s gaze, we asked students to report on their perception of teacher support adaptivity, since it has been found that students can accurately assess the adaptivity of teacher support (Parson et al., 2018; Van de Pol et al., 2022).

## Methods

### Design and participants

This study was a between-subjects experiment with two conditions: gaze display and no gaze display (control). Students were randomly allocated to one of the two conditions using Microsoft Excel (*n*_*control*_ = 24, *n*_*gaze display*_ = 25). Teachers were allocated to conditions based on availability and participated in 1 to 10 different sessions. Seven teachers participated in both conditions. All participants had self-reported normal or corrected-to-normal visual acuity. This study was approved by the Ethics Review Board of the Faculty of Social and Behavioral Sciences of Utrecht University. Sample size planning was based on practical constraints (Lakens, 2022).

### Experimental set-up

**Fig. 2:**
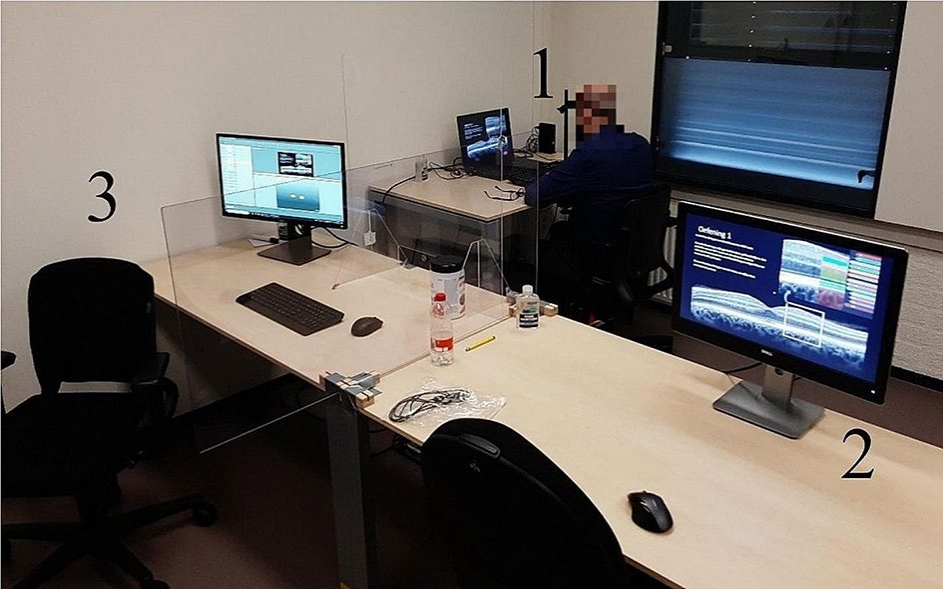
Experimental Set-Up of the Present Study. *Note* (1) The student is sitting behind the laptop with the mobile eye-tracker and a desk with a forehead rest attached. (2) The teacher sits behind a screen that displays what the student sees either with (gaze-display condition) gaze display or without (control condition) gaze display visible. (3) Experimenter desk

The experiment was developed using SMI Experiment Center software (Version 3.7; SensoMotoric Instruments GmbH, 2017) and presented to the students on a laptop screen (Dell Precision M4800; 1920 × 1080 pixels) which subtended 44° of visual angle horizontally and 28° of visual angle vertically at a viewing distance of 59 cm. Eye movements were recorded using an SMI RED250mobile eye-tracker with a sampling rate of 250 Hz (SensoMotoric Instruments GmbH, 2017). They were displayed to teachers on a Full HD 24” monitor (Dell P2417H; 1920 × 1080 pixels) via the SMI Experiment Center Software preview function. Figure 2 provides an overview of the setup.

The web lecture was played on the student’s screen, using speakers as an audio output. A knowledge pre-test and post-test and the questionnaire were administered using Qualtrics software (Version 6.21; https://www.qualtrics.com/) on the same screen.

## Materials and measures

### OCT fundus images

Seventy-five OCT fundus images were selected from the Heidelberg Engineering Academy website (https://www.academy.heidelbergengineering.com). Images were stored as (mostly) monochromatic JPEG images and standardized in size and magnification to fit the screen resolution. Forty-five images were used for the web lecture, 4 images were used for the pre-test, 18 images were used for the one-to-one teaching sessions, and 8 images were used for the post-test. The topics ‘systematically examining an OCT scan’, ‘anatomy of the chorio-retina’, and ‘etiology and stages of ocular pathology’ were covered in both the web lecture and the learning task. The complexity of cases in the learning tasks was higher than in the web lectures, and thus the cases in the post-test were more difficult than in the pretest.

### Weblecture

The web lecture was an eleven-minute instruction video about interpreting OCT images. The students were instructed regarding several anatomical views of the retina and the use of various settings within diagnosing a patient’s retina (physiological vs. pathological) whilst referring to the relevant structures of the retina.

### Performance on pre-test

The pre-test was a computer-based knowledge test comprising 10 multiple-choice questions about the lectures’ content, each with four possible answers (Appendix A). The score was the number of correct answers. The tests were developed by one of the authors and a faculty teacher, both subject matter experts (SMEs) on OCT, who did not participate in this study. During two meetings, the SMEs discussed the multiple-choice questions and reached a consensus on the administered pre-test and post-test.

### Learning tasks for one-to-one teaching sessions

A total of 10 learning tasks with OCT images were presented in SMI Experiment Center to mimic the real-life task of OCT interpretation (Appendix B). These tasks were developed to train the application of the knowledge and procedures learned in the web lecture. The program automatically switched to the next slide after the set time (one to five minutes, depending on the task), but participants could advance earlier if they had finished. The maximum duration for all learning tasks was 20 min. The teacher received an answer sheet that they could use during the teaching session.

### Performance on post-test

Each student carried out a computer-based knowledge test to measure the knowledge gained about interpreting OCT images. The posttest consisted of 10 multiple-choice questions, each with four possible answers (Appendix C). The questions were similar to the pre-test, but their complexity was higher. The score was the number of correct answers.

### Students’ perception of support

It has been found that students can identify the adaptivity of their teachers’ academic support (Parsons et al., 2018; Van de Pol et al., 2022). Thus, to operationalize the adaptivity of support, we measured students’ perception of teacher support adaptivity, using the ‘questionnaire on teacher support adaptivity’ (QTSA) (Van de Pol et al., 2022) that contains 27 items. Item 17: “When I am working on an assignment, the teacher turns away to help other students” was deleted since it did not apply to the current situation. Apart from that, the wording of the items from the original questionnaire was kept. Each item is scored on a 5-point Likert scale ranging from ‘[1]. I don’t agree at all’ to ‘[5]. I totally agree’. The QTSA consists of four subscales. Two scales reflect adaptive behavior: An increase of teacher regulation upon low student understanding (Adaptive + or A+), and a decrease of regulation upon higher understanding (Adaptive- or A-). Two scales reflect non-adaptive behavior: An increase of regulation upon higher understanding (Non-adaptive + or NA+), and a decrease of regulation upon lower understanding (Non-adaptive- or NA-).

### Teachers experienced adaptive support

Teachers rated (from 0 to 10) how well they felt they adaptively supported the students during the learning tasks.

### Procedure

The experiment was performed in individual sessions of approximately 60 min. Teachers involved in the eye-tracking condition received oral instructions from the experiment leader about live dynamic gaze displays before the experiment. After signing the informed consent, the students filled in their age and gender, watched the web lecture, and took the pre-test. Subsequently, a nine-point calibration and a five-point validation procedure for the eye tracker were carried out. Calibration was only accepted with an average error of ≤ 1.0° on each point and repeated if necessary. After that, each student worked on computer-based learning tasks supported by a teacher in a one-to-one teaching session in either the gaze-display or control condition. Finally, the students completed the post-test and the QTSA, and the experimenter debriefed the teacher and students.

### Data analysis

Data were analyzed using JASP version 0.14.0 using Bayesian t-tests. As an alternative for the p-value, we reported the inclusion Bayes factor *BF*_*10.*_ which quantifies the relative support in the data for including condition as a predictor (i.e., for a difference between conditions) versus the null hypothesis (i.e., no difference between conditions). Thus, Bayesian analyses help us determine the effects of gaze displays: If the Bayes factor is greater than 1, there is more evidence that gaze displays are more effective than no gaze displays. If the Bayes factor is smaller than 1, there is more evidence for the null hypothesis that the gaze displays are not more effective than no gaze displays (e.g., Kass & Raftery, 1995). Bayes factors thus quantify how much support there is for or against the effect of the conditions. A Bayes factor of 3, for example, can be interpreted as three times more evidence in the data that there is a difference between conditions. A Bayes factor of 0.33 can be interpreted as three times more evidence that there is no difference between conditions. Since this is the first experiment on the use of live dynamic gaze displays, default non-informative priors were used. Assumptions for each analysis were met unless indicated otherwise in the results section.

## Results

### Baseline information

Baseline information for students can be found in Table 1. A total of 49 optometry students (15 male) and ten optometry teachers (*M*_*age*_ = 41.10, *SD =* 9.13, 4 male) consented to participate in the study, all from the HU University of Applied Sciences Utrecht. They had between 1 and 25 years of teaching experience (*M* = 10.70, *SD* = 8.22). Bayesian t-tests suggest that there are no (clear) differences between the conditions in terms of baseline characteristics.

The McDonald’s omega for the Adaptive- scale was 0.69, for the Adaptive + scale 0.72, for the Non-Adaptive- scale 0.75, and for the Non-Adaptive + scale 0.80, which was > 0.70 for all scales but the Adaptive- scale, and could thus be considered acceptable (Tavakol & Dennick, 2011).

To investigate effects of the gaze displays, we first statistically analyze differences between conditions on students’ posttest score and their perception of support as measured with the QTSA. Due to a small number of involved teachers, we only report descriptives for the teachers’ perception of adaptive support.

**Table 1:** Baseline characteristics (student) by condition

		Eye-tracking condition		Control condition		
		M	SD	M	SD	BF_10_
Teacher Experience (years)		11.22	8.54	14.14	7.24	
Age of Participants (years)		22.42	2.36	23.12	2.60	0.425
Average Duration of the One-to-one Teaching Session (minutes)^1^		16.67	2.02	15.94	1.94	0.560
Accuracy of eye-tracking data (DVA)		0.51	0.16	0.53	0.20	0.292
Pre-test score^2^		5.40	1.23	5.25	1.45	0.304
		*n*	*%*	*n*	*%*	
Gender	Male	10	40.0	5	20.8	1.013
	Female	15	60.0	19	79.2	
Year of study	3	13	52.1%	14	58.3%	0.363
	4	12	48.0%	10	41.7%	

*Note *^1^The maximum duration of exercise was 20 min. ^2^The maximum test score was 10. The pre-test and post-test included different questions

### Effects of condition on posttest score and perception of support

The descriptive statistics for the posttest score and each adaptivity facet can be found in Table 2. Although participants in the eye-tracking condition score somewhat higher on the pretest, there was evidence against a difference between conditions on the pre-test (*BF*_*10*_ = 0.304), see Table 1. Thus, the pre-test score was used as a covariate in the analysis of post-test scores (Leppink et al., 2016). We found evidence against an effect of condition on the post-test score (*BF*_*10*_ = 0.335) with the pre-test score as a covariate: The Bayes Factor shows that it is three times more likely that the gaze displays had no effect on the posttest score than that gaze displays had an effect on the posttest score. Perceived adaptivity of support was very similar between the two conditions: Participants perceived that teacher support was very often increased when they did not understand the task (A+) and decreased when they did understand the task (A-). Non-adaptive support was somewhat less common than adaptive support, and participants in the different conditions did not experience different levels of adaptive and non-adaptive support. As can be seen in Table 2, we found evidence against an effect of condition for all four scales: It is 1.5 to 3.5 times more likely that the gaze displays had no effect on the perceived adaptivity of support than that gaze displays had an effect on the perceived adaptivity of support.

**Table 2:** Means, standard deviations, and inclusion Bayes factors for posttest score and per adaptivity facet

	Eye-tracking condition		Control condition		
	M	SD	M	SD	BF_10_
Posttest score^1^	5.28	1.51	5.58	1.84	
Adaptive + ^2^	4.27	0.45	4.35	0.60	0.478^3^
Adaptive−^2^	3.80	0.50	3.66	0.62	0.388^4^
NonAdaptive + ^2^	2.81	0.51	2.95	0.64	0.66^4^
NonAdaptive−^2^	2.01	0.60	2.10	0.59	0.285^4^

*Note n*_*control*_ = 24, *n*_*gaze display*_ = 25. ^1^The maximum test score was 10. The pre-test and post-test included different questions. ^2^For all adaptivity scales, the minimum is 1, maximum is 5. ^3^*BF*_*10*_ gives the inclusion Bayes factor for the effect of condition in based on a Mann-Whitney-U test as the assumption of normality was violated. ^4^*BF*_*10*_ gives the inclusion Bayes factor for the effect of condition in independent samples t-tests

### Teachers’ experience with adaptive support

Teachers rated (on a scale from 0 to 10) how adaptive they felt their support was in the two conditions. Descriptives can be found in Table 3. Teachers participated in both conditions (*n* = 6), only the eye-tracking condition (*n* = 3) or only the control condition (*n* = 1). The assignment to conditions was based on availability.

Note that only one teacher reported a higher level of adaptivity in the control condition, and one teacher rated their adaptivity equal in both conditions. The other five teachers who taught in both conditions rated their adaptivity 1 or 2 points higher in the eye-tracking condition.

**Table 3:** Teachers’ experienced adaptivity of support by condition

	*n*	M	SD	Min	Max
Eye-tracking condition	9	7.33	0.89	6	9
Control condition	7	6.71	0.76	6	8

## Discussion

The goal of this study was to investigate whether live dynamic gaze displays can improve teachers’ adaptive instructional support to students in one-to-one teaching sessions.

Since gaze displays can visualize covert viewing behavior, they can provide more detailed insight into students’ understanding and potential misconceptions. It was therefore expected that seeing a student’s gaze would help the teacher provide more adaptive instructional support and, by that, foster students’ post-test performance. Although we could not execute statistical analyses due to the low number of teachers, the descriptive information suggests that teachers felt that they taught more adaptively when seeing the gaze displays. However, Bayesian analyses provided some initial evidence that even though teachers reported that they provided more adaptive support in the eye-tracking condition versus the control condition, neither students’ post-test performance nor their perception of the adaptivity of the provided support was different between the two conditions. It was approximately 3 times more likely that the gaze displays had no effect on the posttest score, and 1.5–3.5 times more likely that the gaze displays had no effect on the perceived adaptivity of support than that the gaze displays had an effect on the score and perceived adaptivity of support.

While these findings suggest that the use of live gaze displays is not very effective, further research would be needed to warrant that conclusion and to unpack the cause for the lack of expected benefits. One cause might be that this study was the first to investigate the use of *live dynamic* gaze displays. Live dynamic gaze displays differ from the offline static and dynamic gaze displays investigated in earlier research (Emhardt et al., 2020; Knoop-van Campen et al., 2021; Kok et al., 2023; Špakov et al., 2017). In those studies, the observers were quite good at inferring the performer’s answers, strategies, or certainty from their gaze display, but they could generally take their time to interpret a gaze display and were not forced to interpret the gaze display and immediately act on the information to adapt their teaching. Some teachers mentioned during debriefing that this combination of having to interpret and react almost simultaneously was cognitively demanding. Likewise, while earlier studies showed that people can interpret gaze displays in terms of cognitive processes, teachers still expressed doubt in their interpretations during the debriefing of this study (see also Knoop-van Campen et al., 2021; Kok et al., 2022). They also had to get used to the gaze display. Future research should therefore investigate the effects of training teachers in gaze display interpretation and the longitudinal effects of working with (live) gaze displays.

An alternative explanation for the lack of differences between conditions is that gaze displays might not convey relevant information about learners’ cognitive processes. For example, Kok et al., 2022 found that gaze displays did not provide useful information for students to assess their own learning. In that paper, students practiced navigational map reading tasks. It was found that students interpreted the gaze display in terms of whether they searched for landmarks at the right location. However, students struggled mostly with interpreting the map features. Thus, the gaze displays did not visualize the aspects of the task that students struggled with most (Kok et al., 2022). In our study, however, cases were selected on which students are known to struggle with searching for the correct retinal layers and interpreting pathological information, and thus the gaze display should provide important information for the teacher to base their support on. Further research could investigate how teachers interpret gaze displays, and if and how they would act on them (cf. Knoop-van campen et al., 2021).

Furthermore, an explanation could be that teachers may not have used the cues to make judgments, may not have used judgments to provide adaptive support, or might have used ineffective support strategies. Regarding the first explanation, it could be the case that teachers did not optimally use the performance cues provided by the gaze display, and relied more on student cues (i.e., student characteristics, like their conscientiousness or interest) or their prior performance, as all students and teachers were familiar with each other (Oudman et al., 2018). While performance cues are generally more diagnostic of later performance (and thus more informative for adaptive support), teachers tend to use student cues if those are available and if performance cues are difficult to extract (Oudman et al., 2018; Van de Pol et al., 2021) Thus, our teachers might not have felt the need to use the gaze display as information about students’ understanding. Further research could investigate the effectiveness of gaze displays in situations where teachers and learners do not know each other (and thus, student cues are unavailable). However, the current set-up is most reflective of authentic practice: Learning a complex visual task in one-to-one teaching sessions generally takes place with the same supervisor-student pairing over an extended period. Furthermore, a detailed analysis of student-teacher interaction (e.g., see van de Pol & Elberts, 2013) might provide insights into whether teachers indeed used judgment to provide adaptive support, or whether they might have used ineffective support strategies. Finally, since we measured students’ perceptions of the adaptivity of support, it could be the case that increases in adaptive support might have been too subtle for students to pick up.

### Limitations and future directions

The teachers did not receive targeted instructions about how to approach their teaching during the intervention. There is a large variation in teacher adaptivity (Van de Pol et al., 2015), which is also reflected in the students’ perception of adaptive support. While most teachers seemed to have used adaptive teaching methods, they also applied non-adaptive teaching methods, such as providing more support when students already understood the task. It would be interesting to investigate the added value of gaze displays when training teachers to be adaptive in their support. Furthermore, we did not counterbalance the order in which teachers were assigned to different conditions for practical reasons. The teachers who taught in more than one condition could thus have applied knowledge gained in the other condition. However, we do not consider this likely, as we expect that the gaze display mostly supports in-the-moment adaptive decisions. Further research could control assignment of teachers to conditions to avoid spill-over effects.

A second limitation is that we investigated the effect of the intervention only on an immediate posttest and long-term retention of skills was not investigated. In line with our earlier recommendation to investigate the usefulness of gaze displays to support adaptive teaching over an extended period of time, it would also be interesting to investigate retention of skills.

A strength of the current experiment is the use of Bayesian analyses to quantify the evidence that there was no effect of the gaze displays on learning or perceived adaptivity of support. However, even though both the mean posttest score and the mean perceived adaptivity of support were very similar in the two conditions, we found relatively weak evidence for the null hypothesis. This was due to the small sample size. As sample sizes increase, the evidence, whether for or against the effect of gaze displays, tends to strengthen (Brysbaert, 2019).

It is important to note that finding evidence to support the idea that there is no effect of gaze displays (favoring the null hypothesis) typically necessitates about three times more participants than finding evidence supporting the idea that there is an effect (Brysbaert, 2019). But even though the evidence against an effect of condition (i.e., favoring the null hypothesis) was not very strong in our data, it is still about three times stronger than the evidence in favor of a difference between conditions.

Given the laborious nature of this experiment, including a larger sample was not feasible. However, an important advantage of Bayesian analyses is that follow-up experiments can build on the current findings to use informative priors (Kruschke & Liddell, 2018).

## Conclusion

We investigated whether live dynamic student-to-teacher gaze displays (i.e., visualization of eye-tracking data) could help teachers to provide more adaptive instructional support and, thereby, impact students’ learning. Bayesian analyses provide some initial evidence that live dynamic gaze displays did not help teachers to teach more adaptively and improve students’ learning. Interestingly, descriptive statistics suggest that teachers nevertheless did feel that they taught more adaptively when seeing the gaze displays. However, we could not execute statistical analyses due to a low number of participating teachers. Further research is necessary to investigate if live dynamic gaze displays can impact adaptive teaching over longer periods or with more teacher training. If so, this could support teachers to be more adaptive not just in regular one-to-one session but also in distance teaching.

## Electronic supplementary material

Below is the link to the electronic supplementary material.

Supplementary Material 1
