# Supplementary material for: Teaching through their eyes: effects on optometry teachers’ adaptivity and students’ learning when teachers see students’ gaze
Source: Adv Health Sci Educ Theory Pract. 2024 Apr 10;29(5):1735–48. doi: 10.1007/s10459-024-10325-3 (PMC11549187; doi:10.1007/s10459-024-10325-3)
Supplement: Supplementary file 1 — Supplementary Material 1 [file 10459_2024_10325_MOESM1_ESM.pdf]

Article title: Teaching Through Their Eyes: Optometry Teachers' Adaptivity and Students' Learning When Teachers See Students' Gaze

Journal name: Advances in Health Sciences Education

Authors: Robert-Jan Korteland, Ellen Kok, Casper Hulshof & Tamara van Gog

Correspondence concerning this manuscript should be addressed to Dr. Ellen Kok, Department of Education, Utrecht University, P.O. Box 80140, 3508 CS, Utrecht, The Netherlands. E: e.m.kok@uu.nl

Appendices

Appendix A: Pre-test (translated from Dutch)

1. The absorption of infrared light causes a dark spot around the macula. There are several possible causes for such abnormalities in the retina. Which structure does not cause a dark spot around the macula?

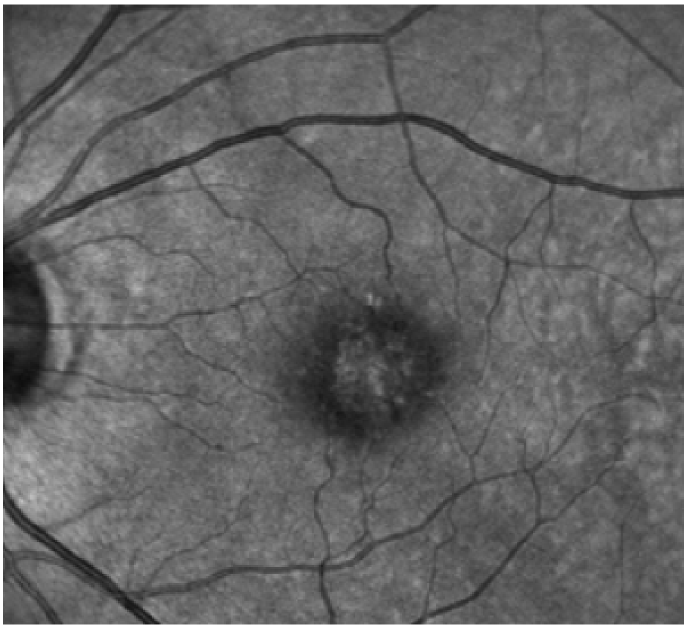

a) Intraretinal fluid
b) Pigment changes in the RPE
c) Intraretinal exudates
d) Subretinal fluid

2. The steps for examining an OCT scan are:
a) Assess scan quality; grade entire scan profile; evaluate foveal profile; identify foveal cross section; assess the different retinal layers
b) Assess scan quality; grade entire scan profile; identify foveal cross section; evaluate foveal profile; assess the different retinal layers
c) Grade entire scan profile; assess scan quality; evaluate foveal profile; identify foveal cross section; assess the different retinal layers
d) Assess scan quality; identify foveal cross section; grade entire scan profile; evaluate foveal profile; assess the different retinal layers

3. During the last step of the OCT-routine, the transitions between the retinal layers must be assessed. Always start with:
a) The retinal pigment epithelium
b) The vitreous body
c) The macula
d) The lamellar structures

4. Sometimes normal retinal anatomic structures cause artifacts in the OCT cross section scan. Which is an example of such a retinal structure?
a) Retinal pigment
b) Retinal blood vessel
c) Retinal nerve fibers
d) Retinal leakage

5. A normal foveal profile in the OCT image has a somewhat straight shape at the level of:
a) The outer retinal layers
b) The inner retinal band
c) The inner retinal layers
d) The outer retinal band

6. The cross-sectional image of the fovea is good when:
a) The retinal pigment layer and the inner plexiform layer intersect in the middle of the fovea
b) The nerve fiber layer and the outer plexiform layer intersect in the middle of the fovea
c) The retinal pigment layer and the middle plexiform layer intersect in the middle of the fovea
d) The nerve fiber layer and the inner plexiform layer intersect in the middle of the fovea

7. The location where the OCT scan should be performed is best determined by means of:
a) Time domain OCT scan
b) 30° HR line scan
c) Fundus photograph
d) Cross sectional OCT scan

8. Which retinal layer represents the red line?

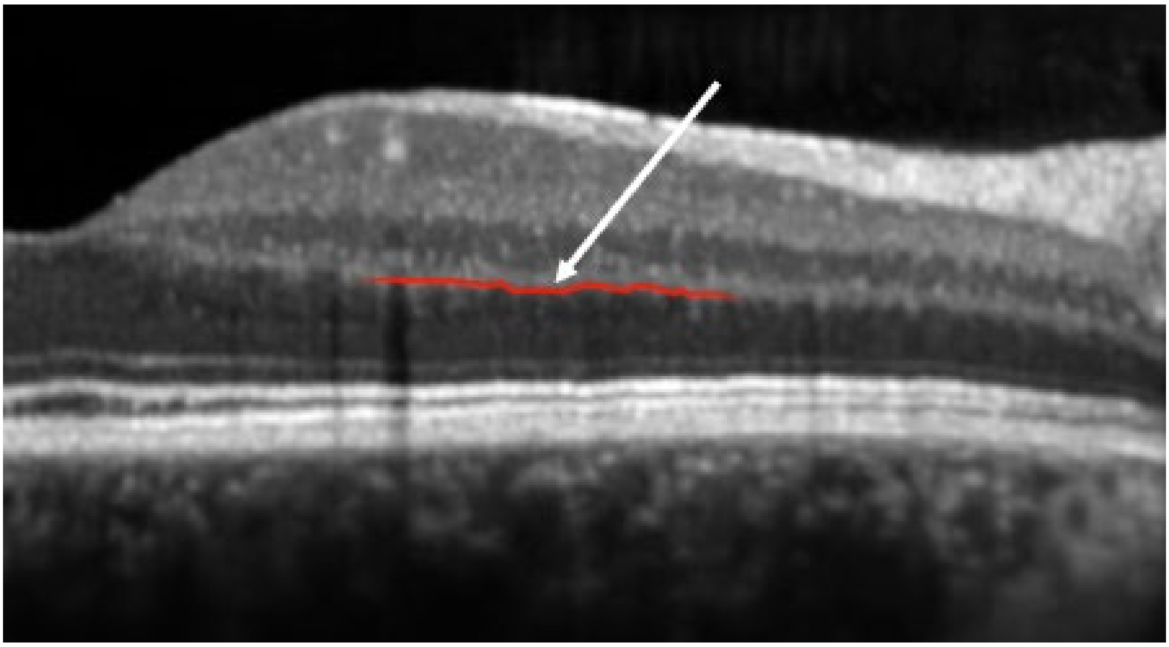

a) Henle's fiber layer
b) External limiting membrane
c) Outer plexiform layer
d) Middle limiting membrane

9. In this OCT cross section photograph, hyperreflective dots can be seen (white arrows). These are

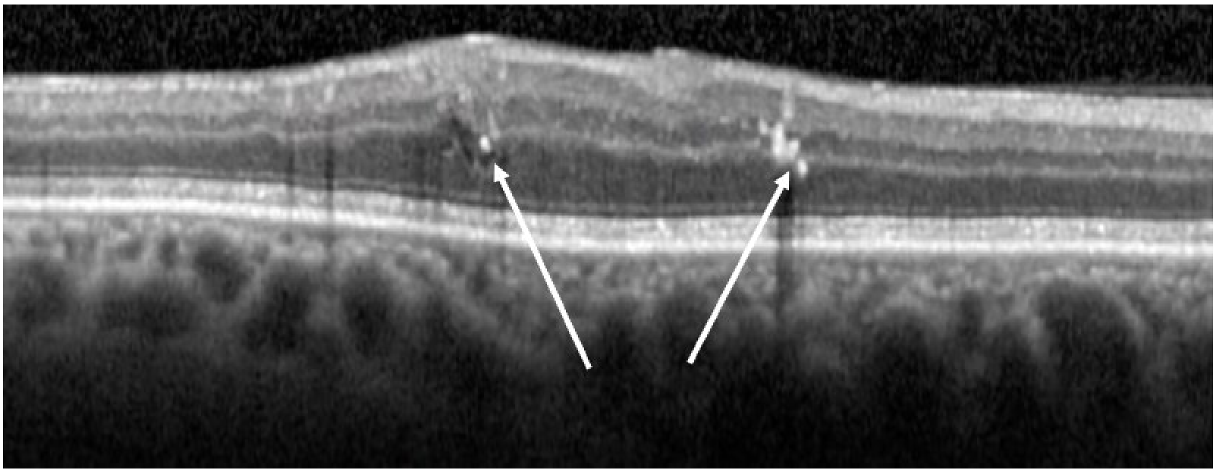

a. Blood vessels
b. Exudates
c. Cysts
d. Drusen

10. The structures of the inner retinal layer are:

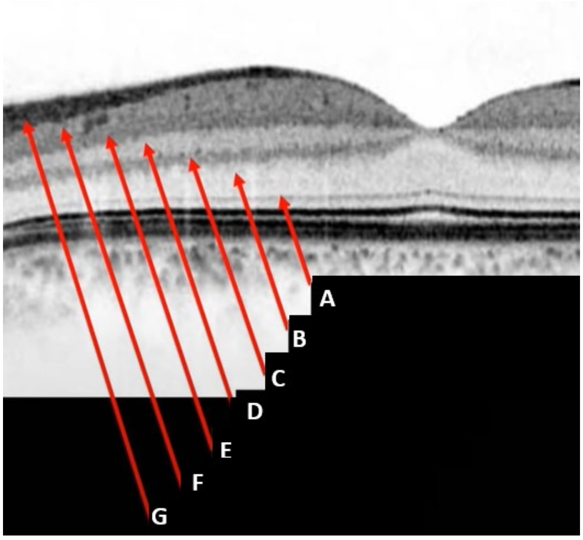

a. A = Retinal Nerve Fiber Layer
   B = Outer Nuclear Layer
   C = Outer Plexiform Layer
   D = Inner Nuclear Layer
   E = Inner Plexiform Layer
   F = Ganglion Cell Layer
   G = External Limiting Membrane

b. A = External Limiting Membrane
   B = Outer Nuclear Layer
   C = Outer Plexiform Layer
   D = Inner Plexiform Layer
   E = Inner Nuclear Layer
   F = Ganglion Cell Layer
   G = Retinal Nerve Fiber Layer

c. A = External Limiting Membrane
   B = Outer Nuclear Layer
   C = Outer Plexiform Layer
   D = Inner Nuclear Layer
   E = Inner Plexiform Layer
   F = Ganglion Cell Layer
   G = Retinal Nerve Fiber Layer

d. A = External Limiting Membrane
   B = Outer Plexiform Layer
   C = Outer Nuclear Layer
   D = Inner Nuclear Layer
   E = Inner Plexiform Layer
   F = Ganglion Cell Layer
   G = Retinal Nerve Fiber Layer

Appendix B: Teacher-supported Learning Tasks (Including Teacher Answer Sheet)

(translated from Dutch)

Exercises

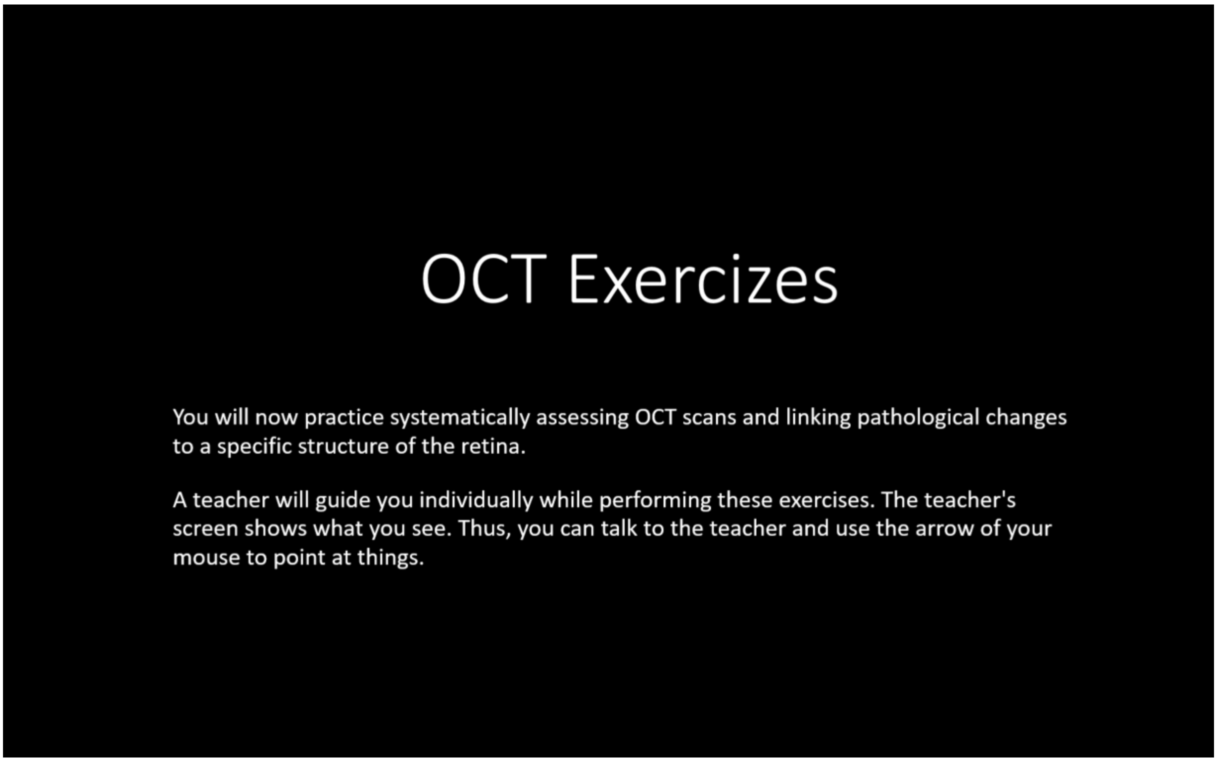

Exercise 1:

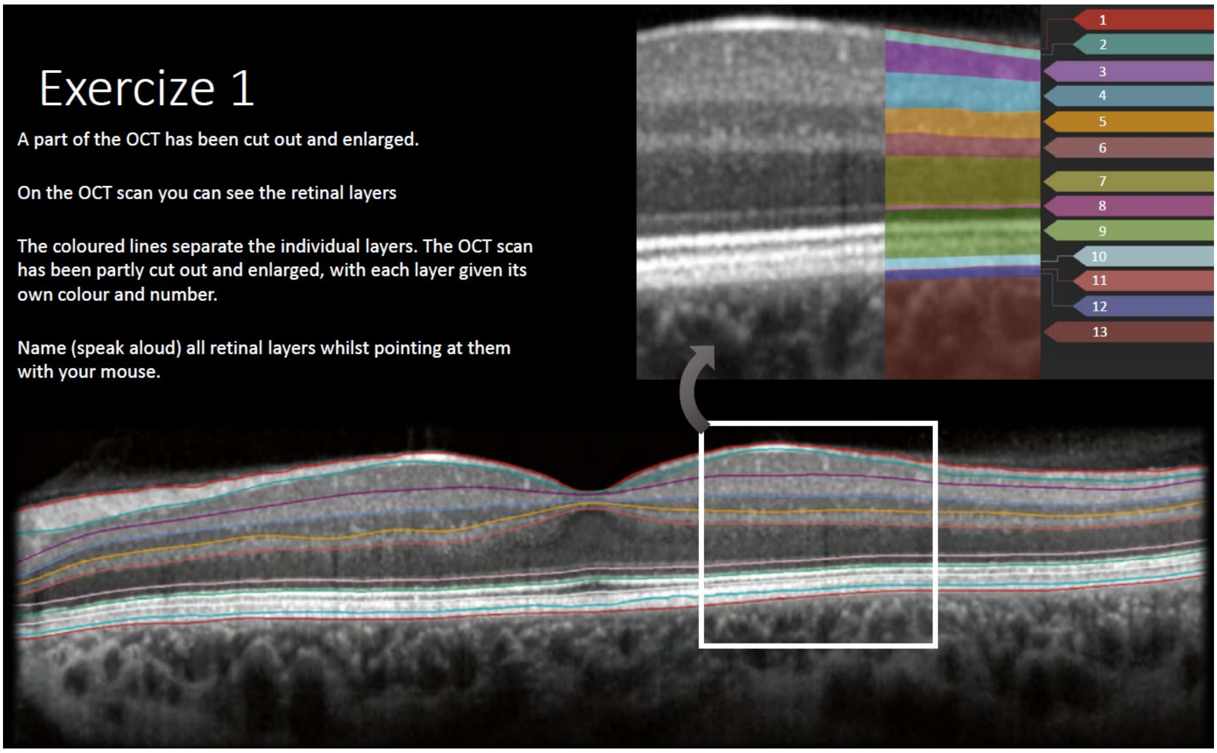

1. Internal Limiting Membrane (ILM)
2. Retinal Nerve Fiber Layer (RNFL)
3. Ganglion Cell Layer (GCL)
4. Inner Plexiform Layer
5. Inner Nuclear Layer
6. Outer Plexiform Layer (OPL)
7. Henle's Fiber Layer (HFL) & Outer Nuclear Layer (ONL)
8. External Limiting Membrane (ELM)
9. Layer of Inner Segments and Outer Segments
10. Retinal Pigment Epithelium (RPE)
11. Bruch's Membrane (BM)
12. Choriocapillaris (CC)
13. (Medium and large) Choroidale Vessels (CV)

Exercise 2:

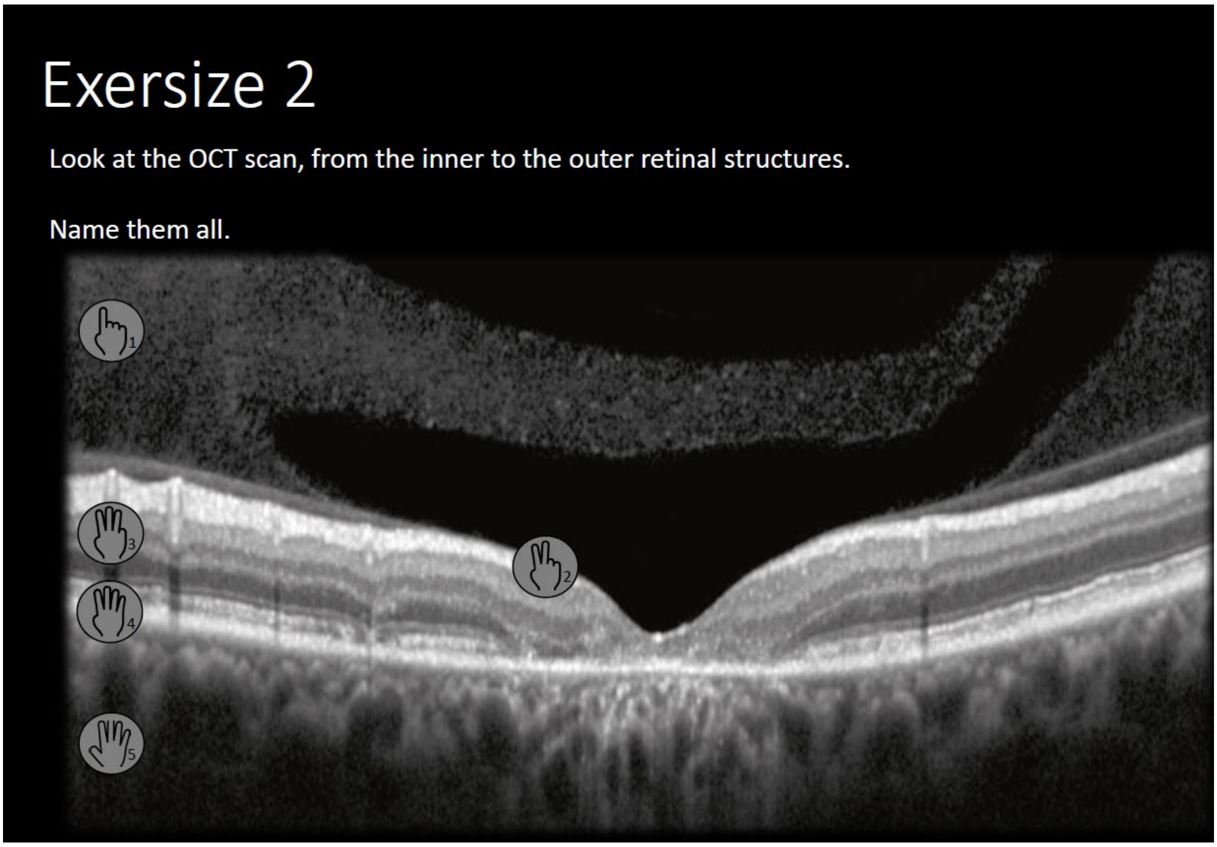

1. Preretinal cavity
2. Foveal configuration
3. Inner retinal layer
4. Outer retinal layer
5. Sub-neuroretinal/ sub-RPE cavity

Exercise 3:

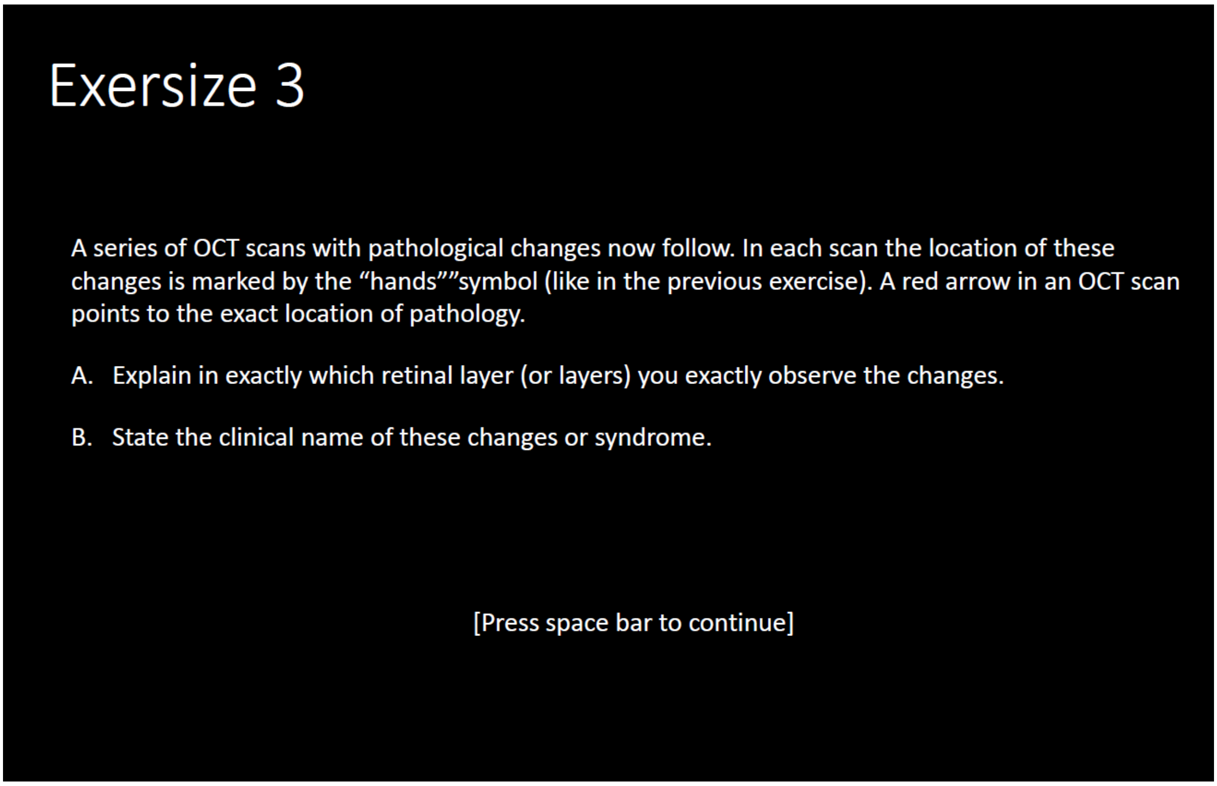

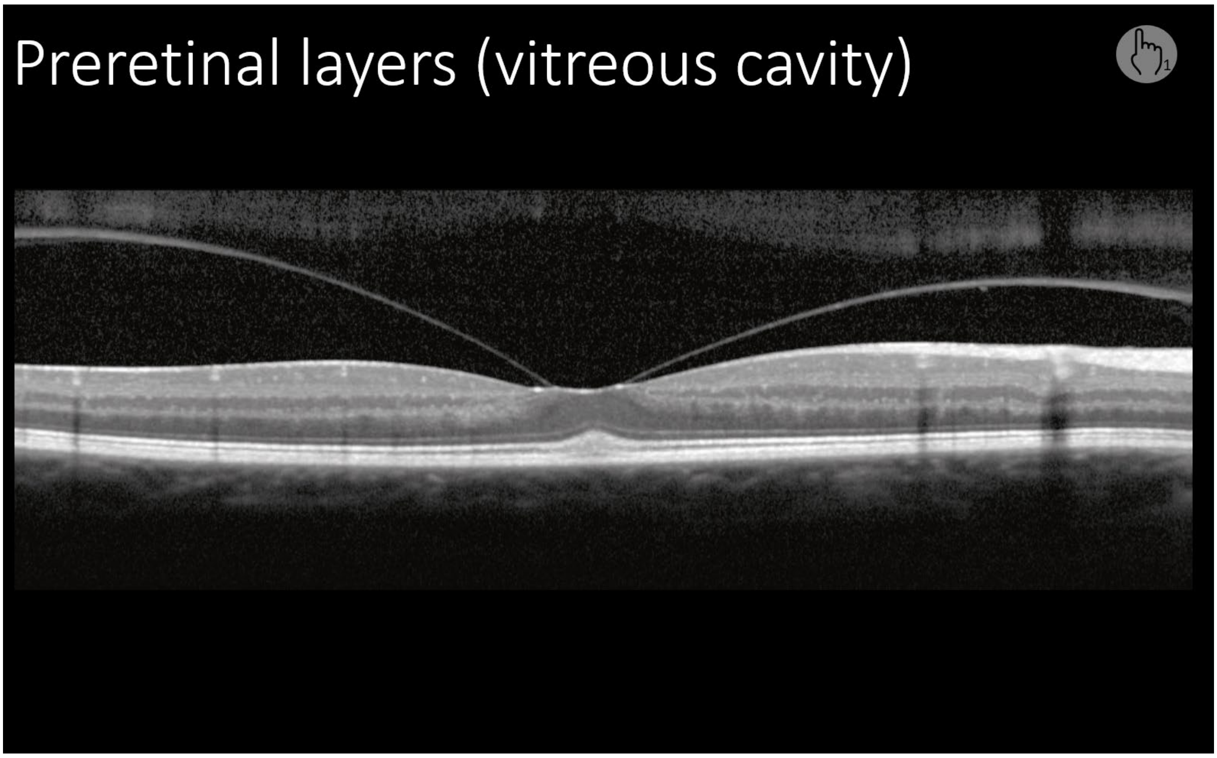

Vitreomacular traction (VMT)

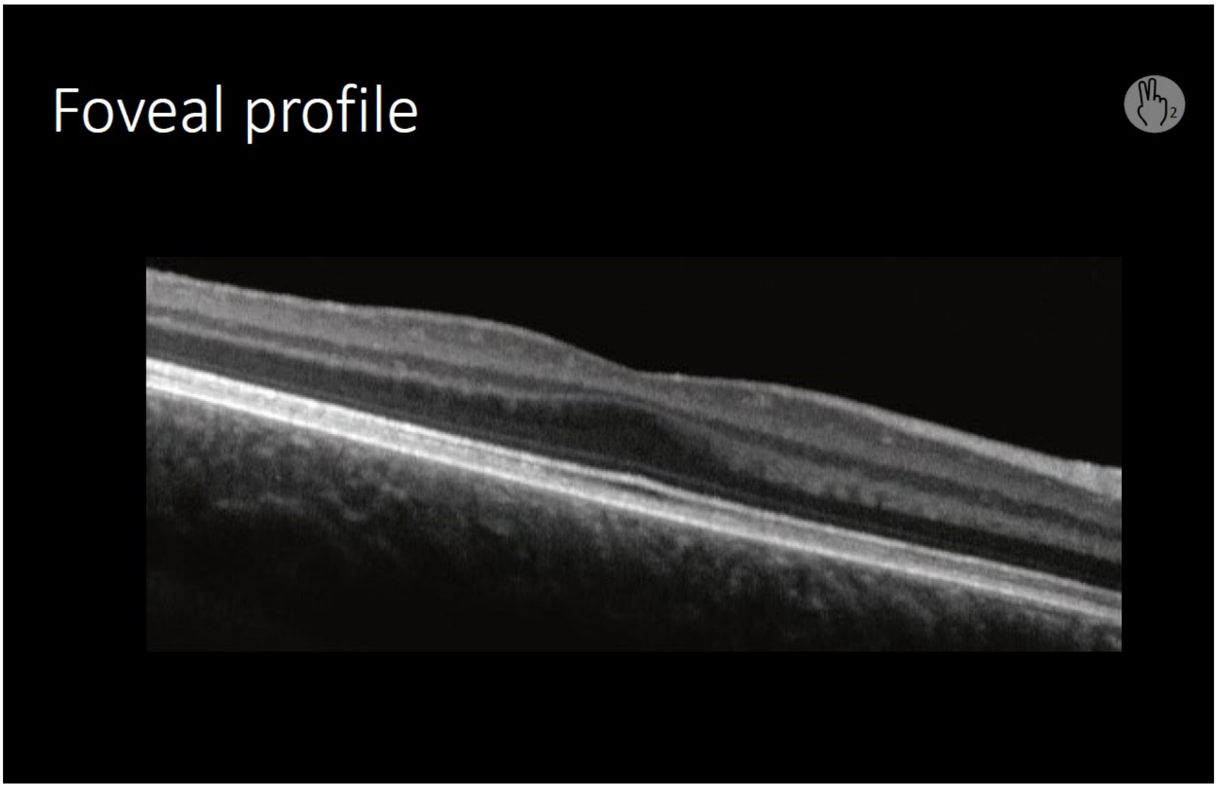

Foveal dysplasia

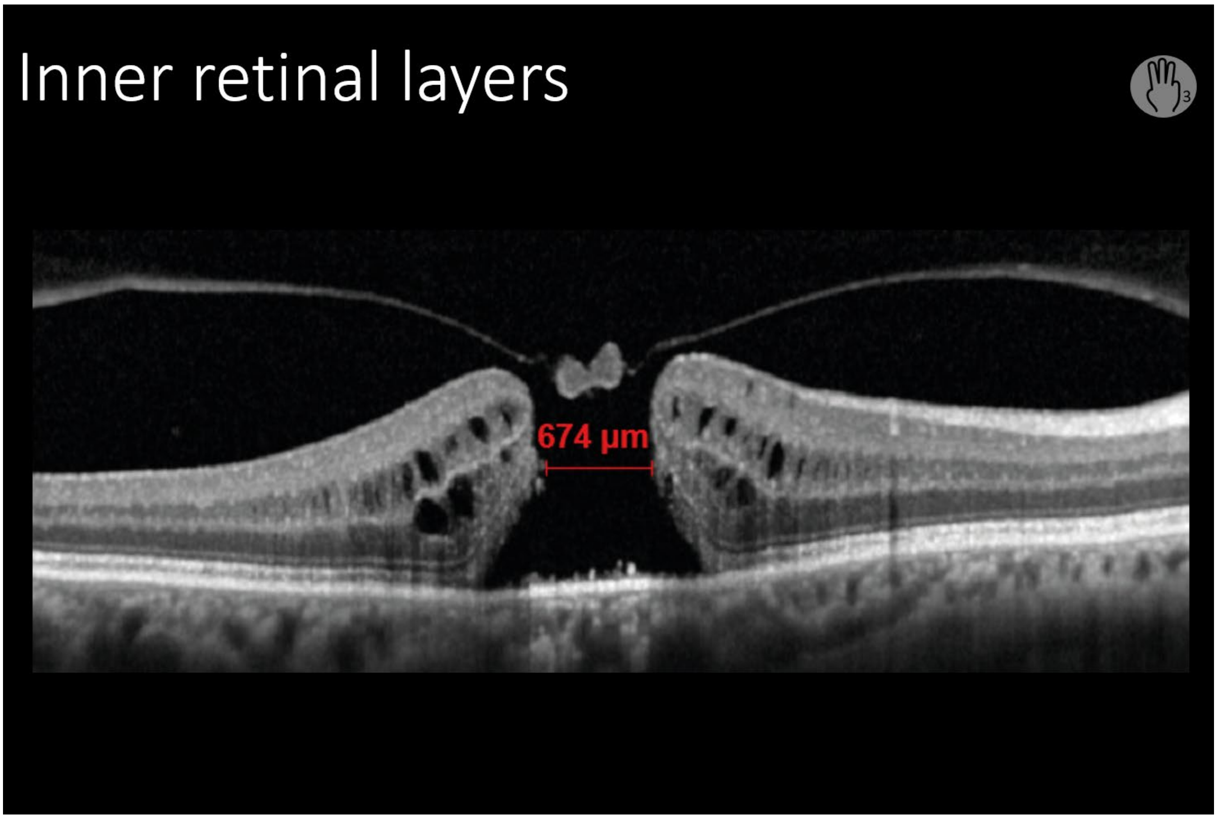

Full-thickness macular hole: Showing the characteristic complete interruption of all retinal layers from Internal limiting membrane to the pigment epithelium. This can also be seen on this OCT scan!

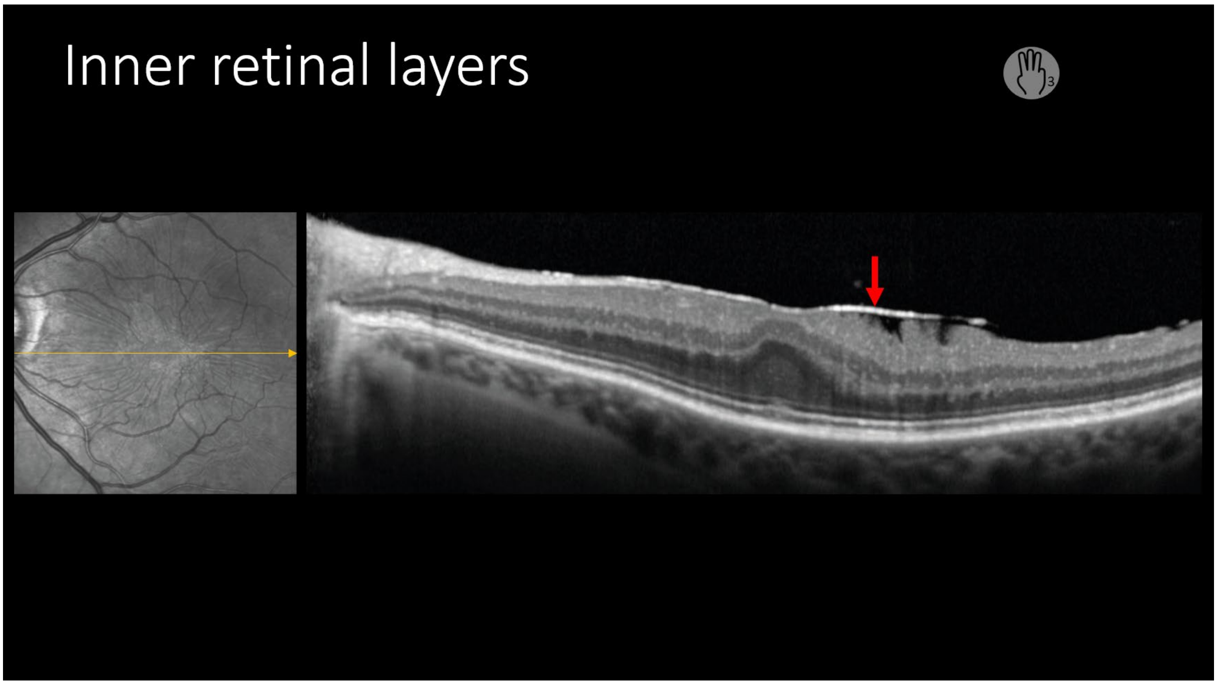

Macular pucker (puckering of the ILM)

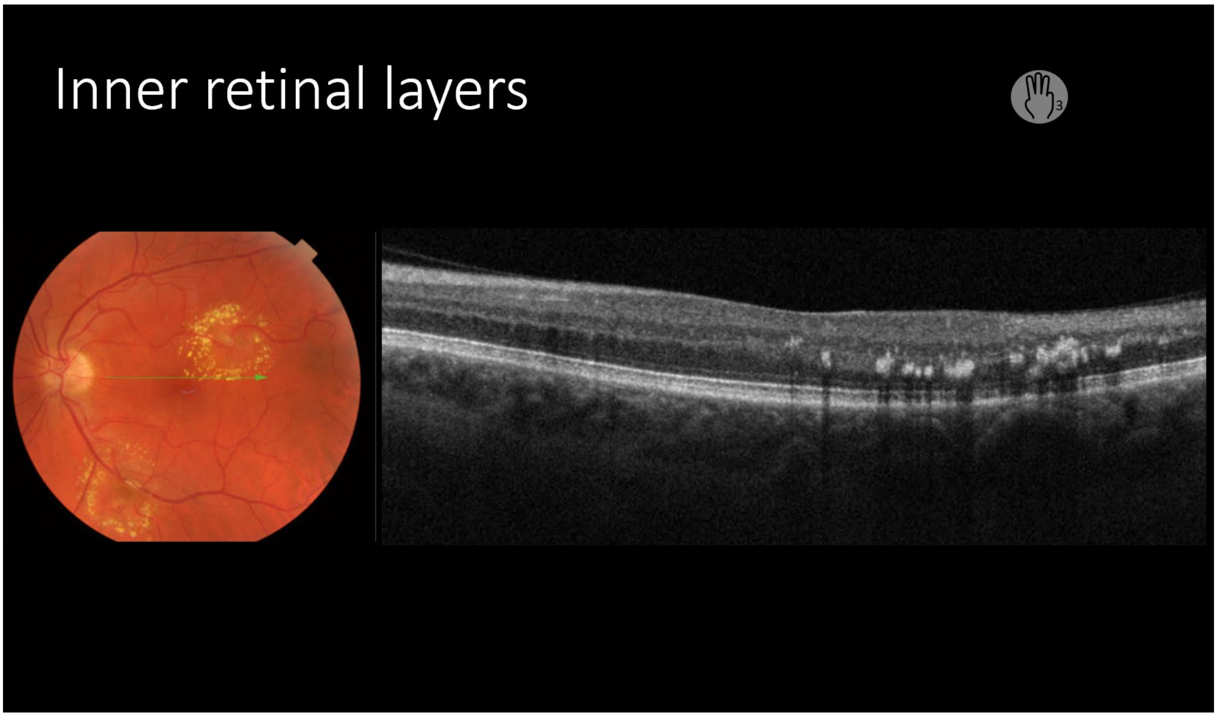

Fundus photograph: Ring-shaped exudates in OS and microaneurysmata. OCT scan exudates (i.e., exudative deposits) near the outer plexiform layer

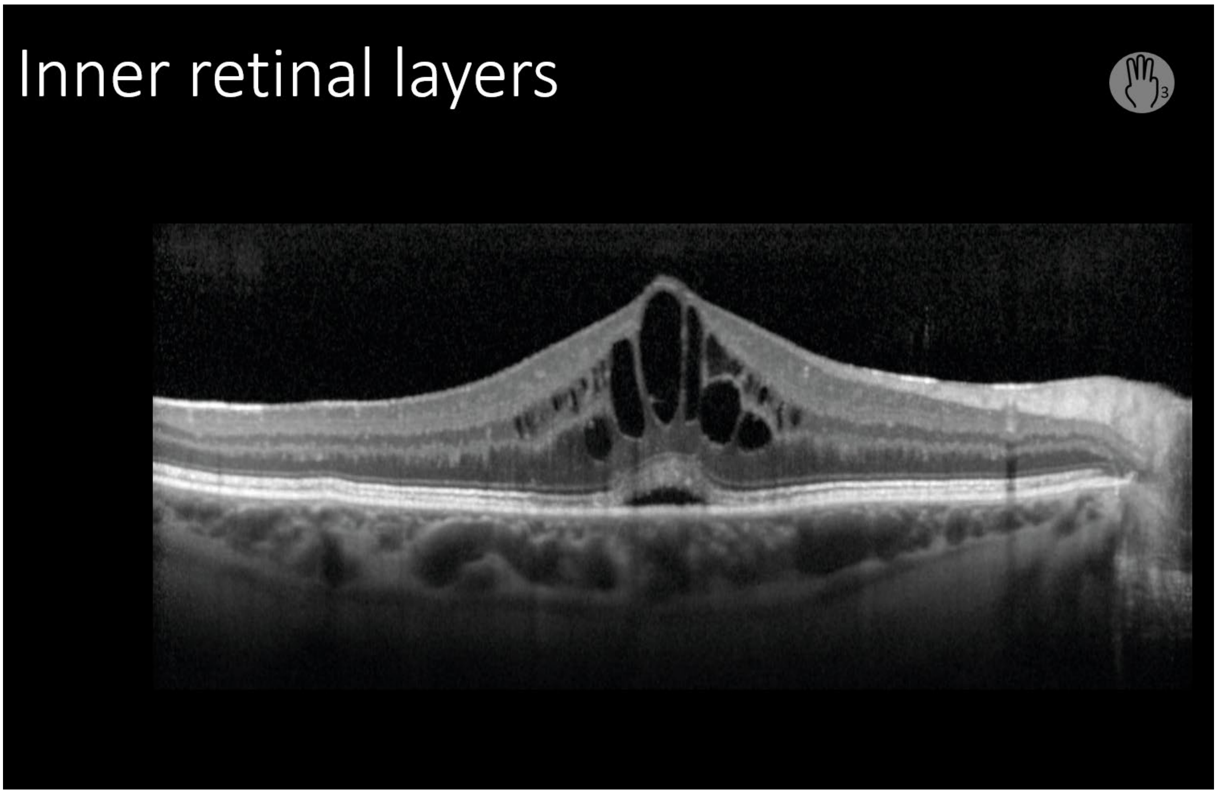

Depending on the (systemic) pathology, intraretinal fluid can occur in several retinal layers. On this OCT scan you can see [diabetic] macular edema, in which cysts are visible from the inner nuclear layer to Henle’s fiber layer (anterior).

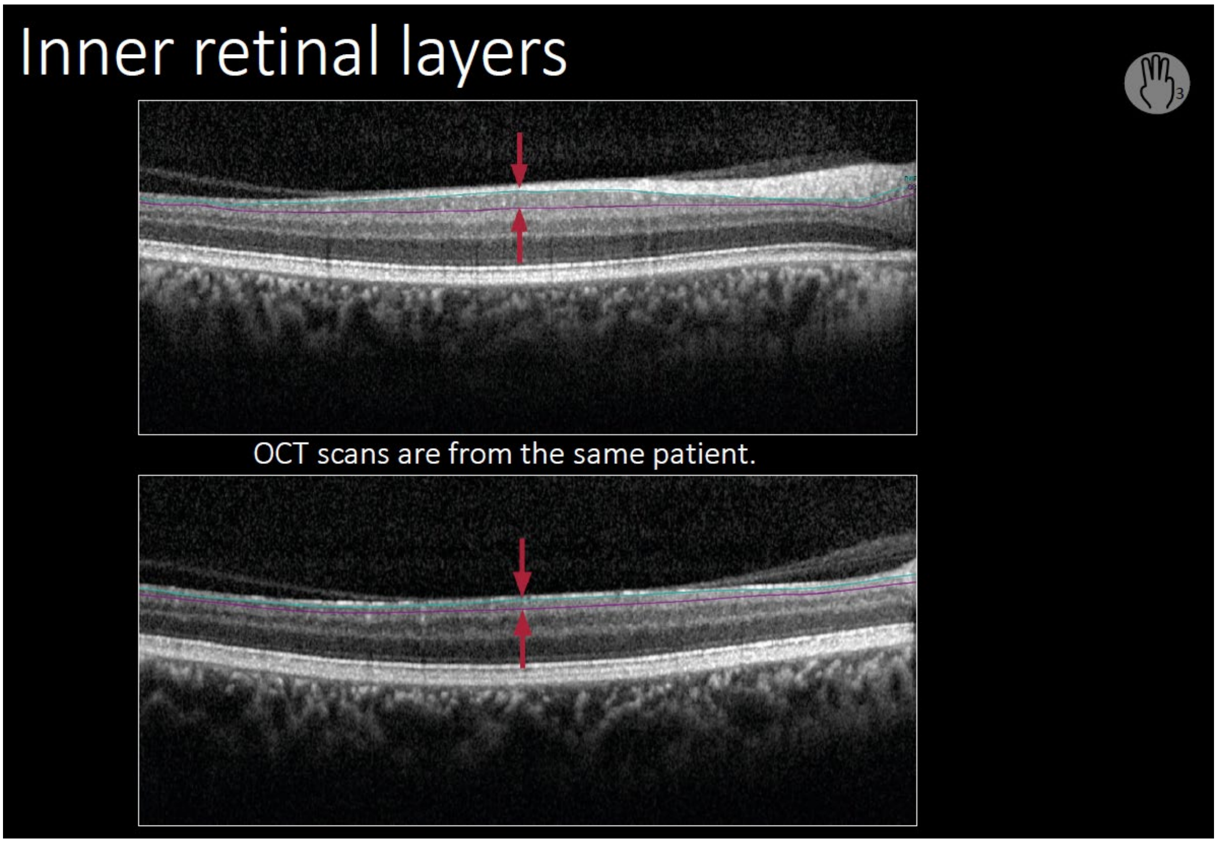

Thinning of the ganglion cell layer (GCL), Ddx glaucoma.

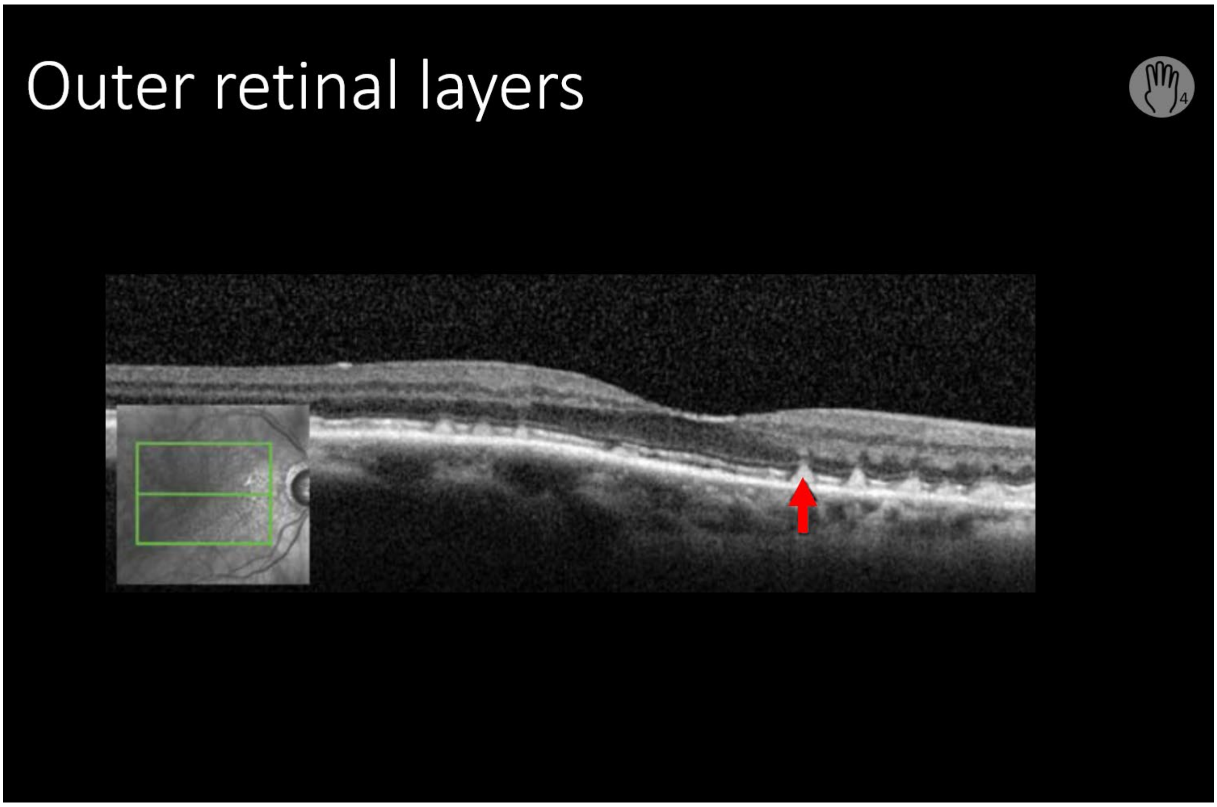

Pseudodrusen (within retina and RPE). Different in appearance than typical drusen in which the location is sub-RPE!

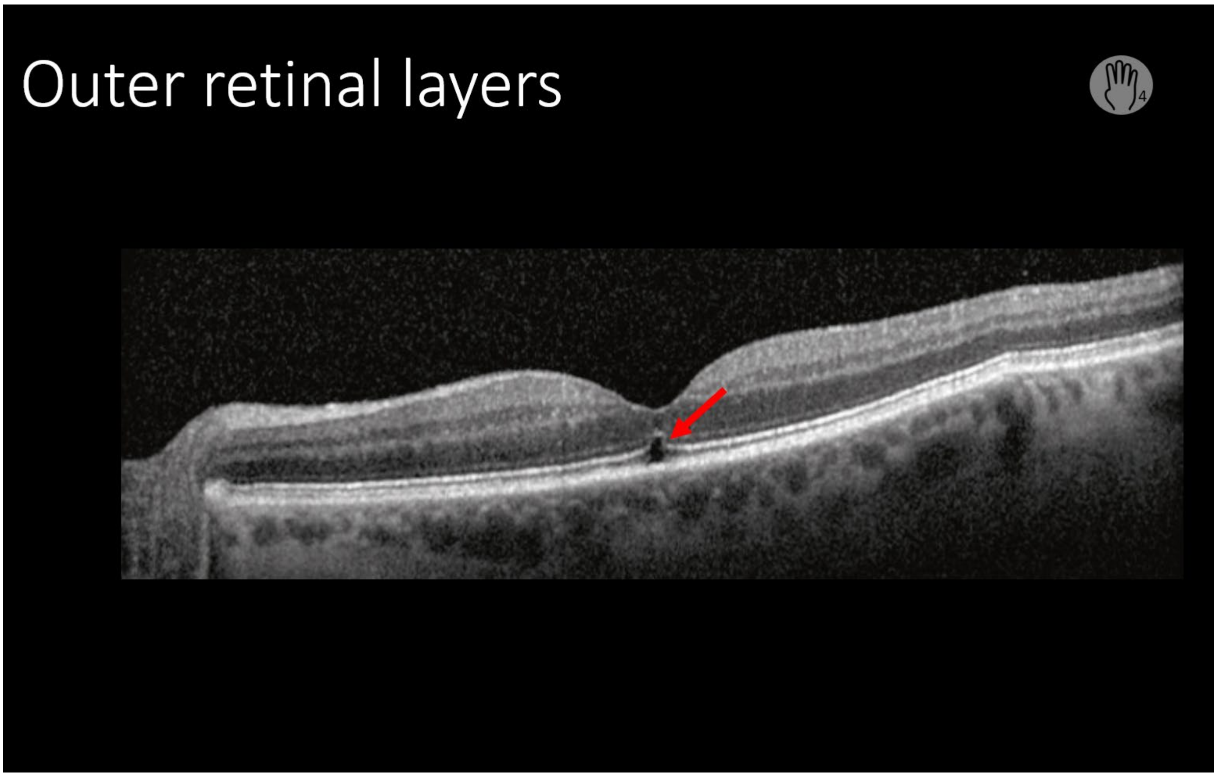

Interruption of the photoreceptor layer. The red arrow points at the the external limiting membrane. The ELM is the boundary between the inner and outer retinal layers. The criterion for inspection of ELM is that the retinal bands behind (posterior) the ELM show 'continuity and integrity'.

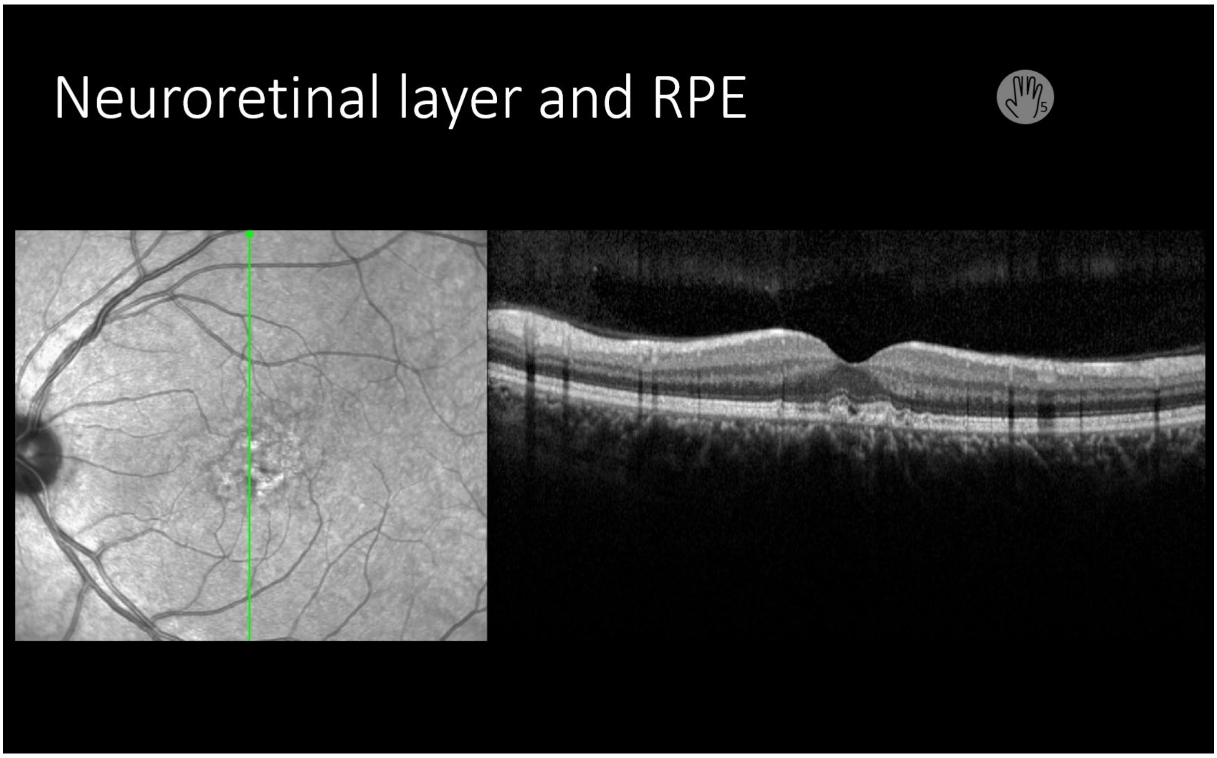

Hard drusen area: ‘Thick dots or bumps’ in the RPE band. Bruch’s membrane is recognizable on the OCT scan as ‘hyper-reflective structure’ <u>beneath</u> the drusen (!)

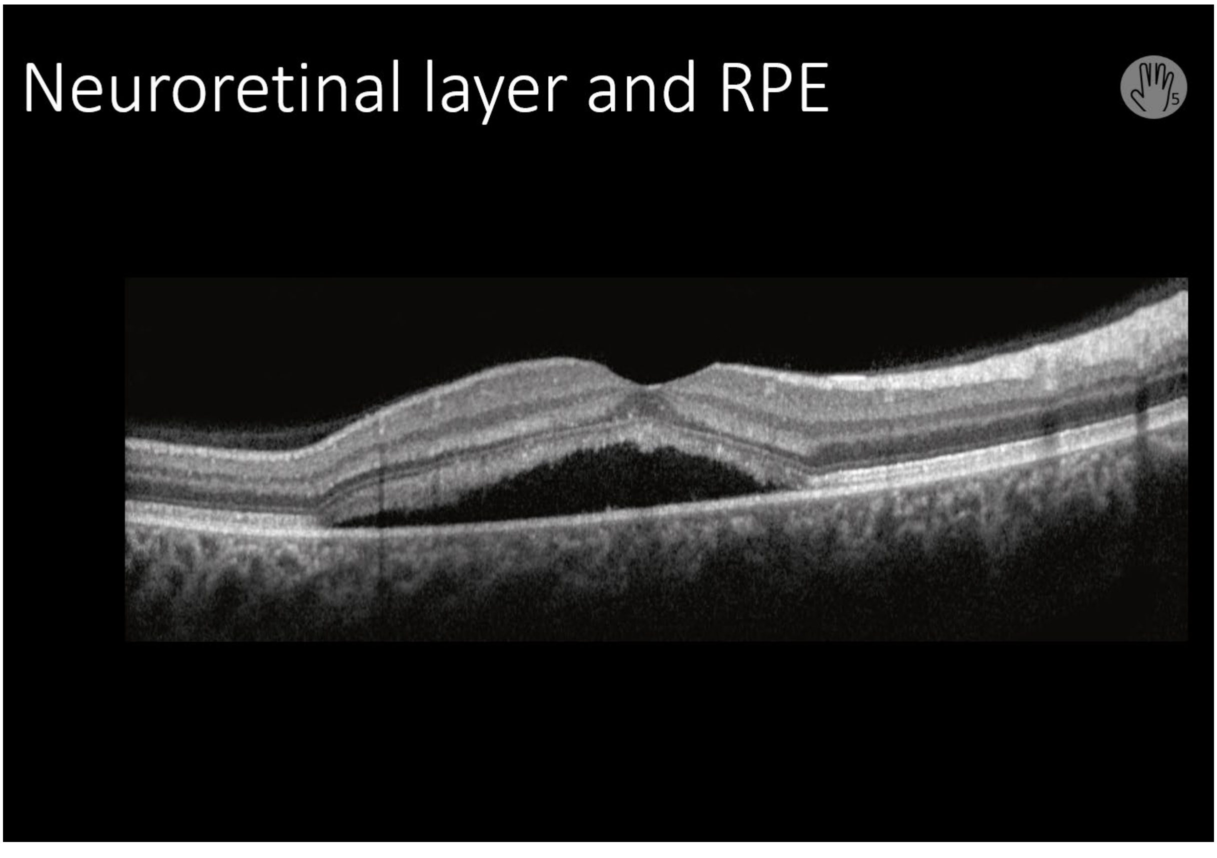

Rupture (detachment) of the neurosensory layer. The thickening of the photoreceptor layer is characteristic.

Appendix C: Post-test

Posttest

1. Which retinal layer does arrow 11 refer to?

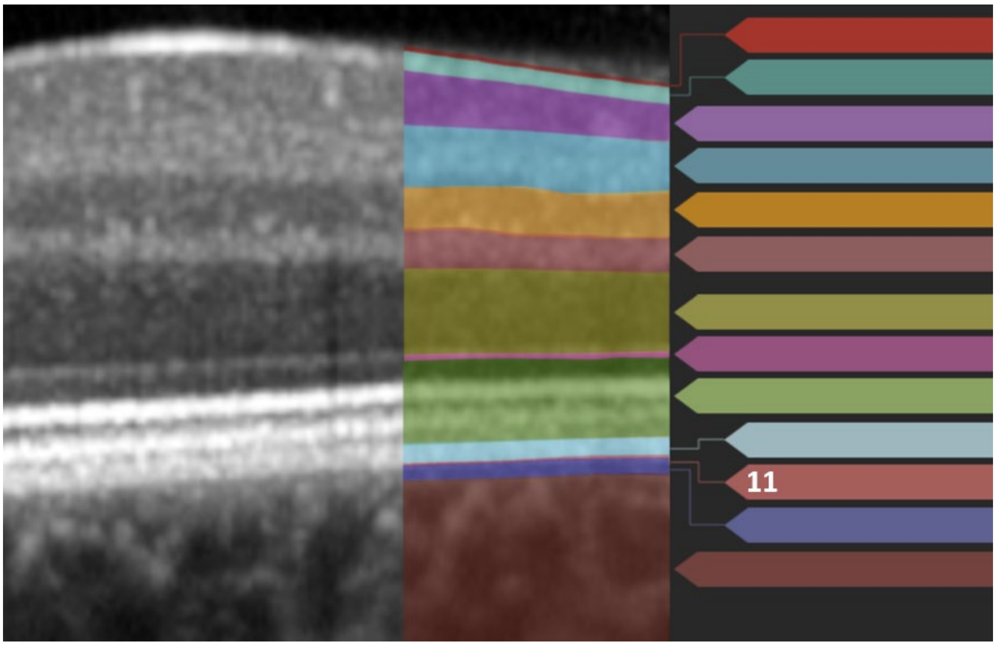

a) Photoreceptor layer
b) Ganglion cel layer
c) Bruch's membrane
d) Inner nuclear layer

2. In which retinal layer are exudates detectable?
a) Outer plexiform layer
b) Outer nuclear layer
c) Inner plexiform layer
d) Inner nuclear layer

3. The fundus photograph shows a typical 'puckering' in the macular area. In which retinal layer do you expect to see this puckering on an OCT scan?

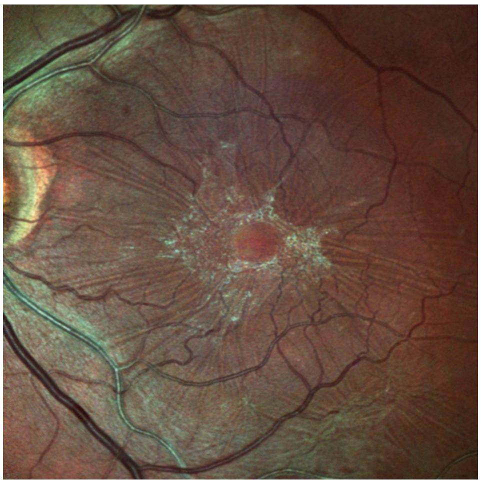

a) Nerve fiber layer
b) Internal limiting membrane
c) Inner nuclear layer
d) External limiting membrane

4. Between which retinal layers can you detect drusen on an OCT scan?
a) Retinal pigment epithelium and Bruch's membrane
b) Inner plexiform layer and outer plexiform layer
c) Photoreceptor layers and retinal pigment epithelium
d) Inner nuclear layer and inner plexiform layer

5. Which retinal structure is indicated by 'hand 4'?

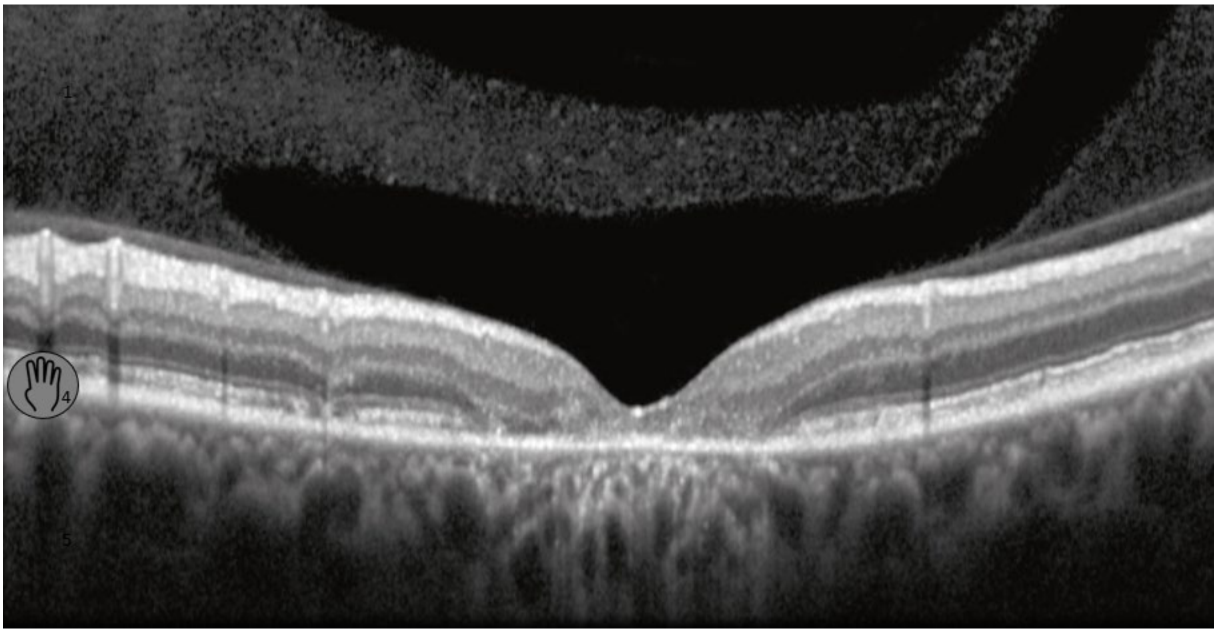

a) Preretinal cavity
b) Inner retinal layer
c) Sub-neuroretinal/ sub-RPE cavity
d) Outer retinal layer

6. Which disorder in the Internal limiting membrane (ILM) is visible in this image?

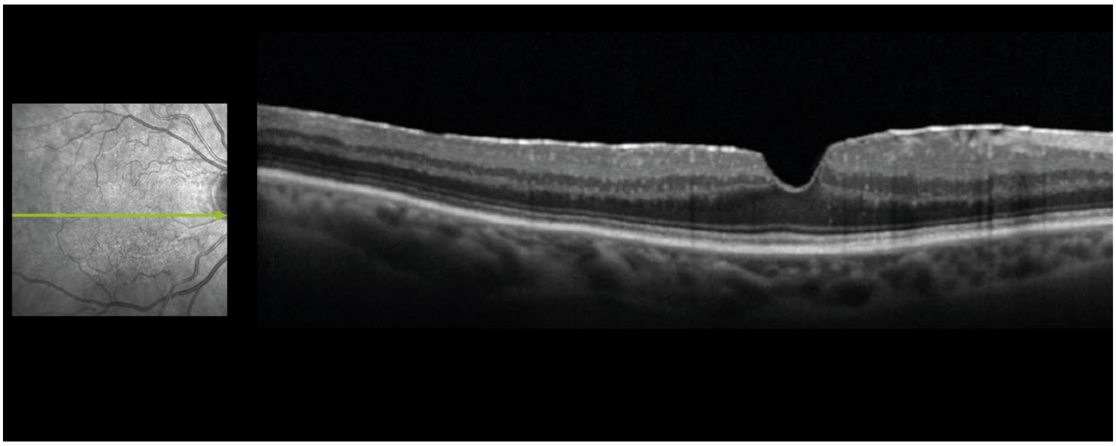

a) Lamellar macular hole
b) Macular edema
c) Vitreomacular adhesion
d) Macular hole

7. On this OCT scan, retinal fluid and drusenoid deposits are visible. Which diagnosis is associated with this clinical picture?
a) Full Thickness Macular Hole (FTMH)
b) Central Serous Chorioretinopathy (CSR)
c) Pigment Epithelial Detachment (PED)
d) Diabetic Macular Edema (DME)

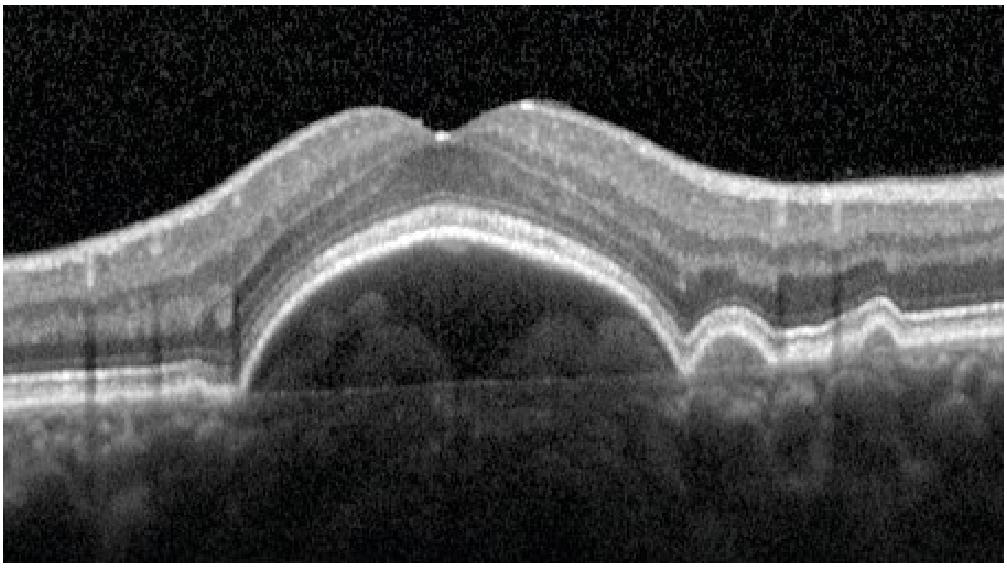

8. Look at the infrared fundus image and the OCT cross-sectional scan. Which of the following is the cause of the shadow where the line is visible on the OCT scan?

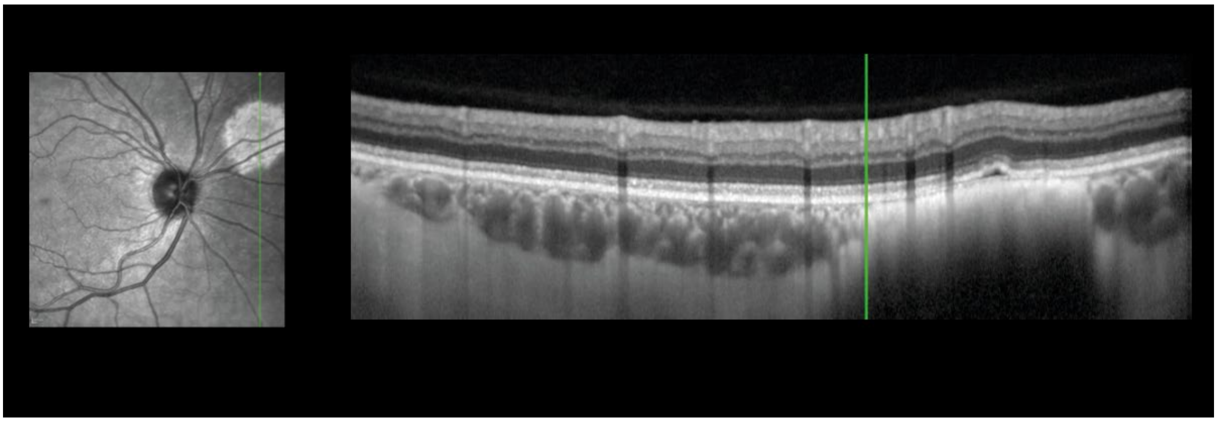

a) Retinal blood vessel
b) Choroidal naevus
c) Papil edema
d) Scar tissue

9. Which retinal layer does the red arrow refer to?

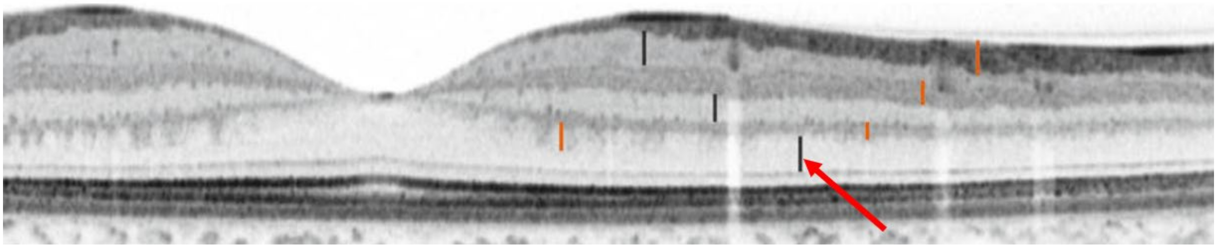

a) Ganglion cell layer
b) Outer nuclear layer
c) External limiting membrane
d) Inner nuclear layer

10. On this OCT scan oval hyporeflective structures are visible surrounded by a hyperreflective band. In which retinal layer do these changes take place?

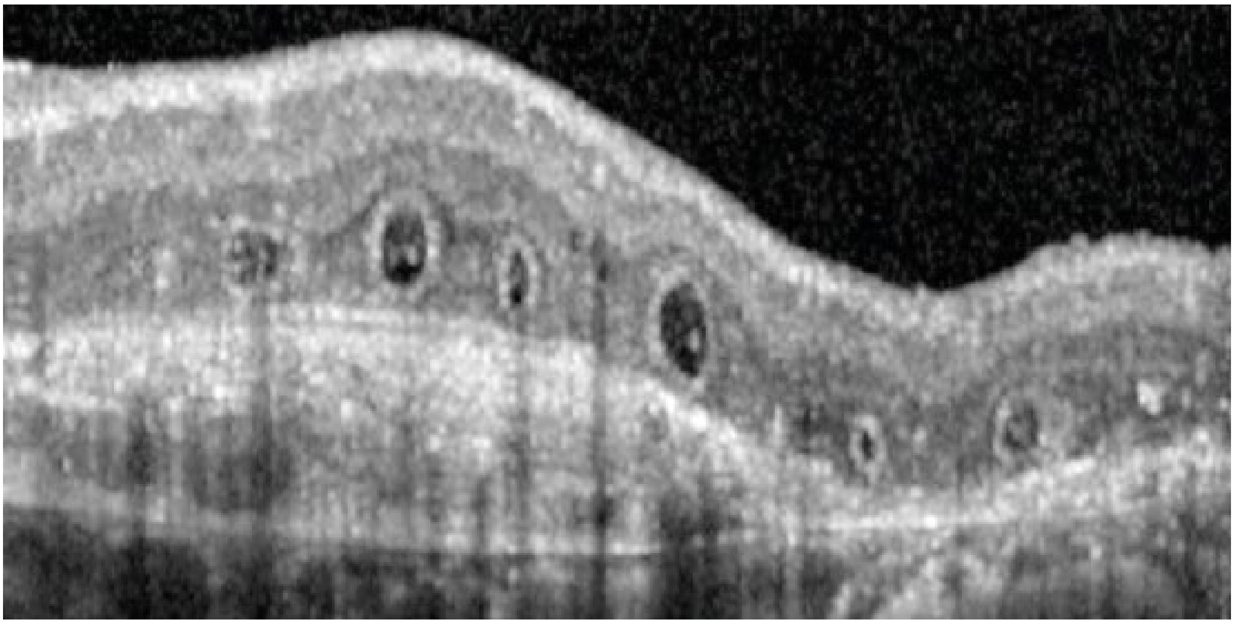

a) Outer nuclear layer
b) Inner nuclear layer
c) Photo receptor layer
d) Bruch’s membrane
